# Screen-Printed Carbon Electrodes Modified by Rhodium Dioxide and Glucose Dehydrogenase

**DOI:** 10.4061/2010/324184

**Published:** 2011-03-03

**Authors:** Vojtěch Polan, Jan Soukup, Karel Vytřas

**Affiliations:** Department of Analytical Chemistry, Faculty of Chemical Technology, University of Pardubice, Studentská 573, 532 10 Pardubice, Czech Republic

## Abstract

The described glucose biosensor is based on a screen-printed carbon electrode (SPCE) modified by rhodium dioxide, which functions as a mediator. The electrode is further modified by the enzyme glucose dehydrogenase, which is immobilized on the electrode's surface through electropolymerization with *m*-phenylenediamine. The enzyme biosensor was optimized and tested in model glucose samples. The biosensor showed a linear range of 500–5000 mg L^−1^ of glucose with a detection limit of 210 mg L^−1^ (established as 3**σ**) and response time of 39 s. When compared with similar glucose biosensors based on glucose oxidase, the main advantage is that neither ascorbic and uric acids nor paracetamol interfere measurements with this biosensor at selected potentials.

## 1. Introduction

There exists today an ever-increasing demand for fast, selective, reliable, and, above all, inexpensive analytical methods. For food products, it is necessary to monitor whether or not microbial, or some other form of, contamination has occurred. Furthermore, it is necessary to monitor compliance with given technological procedures and whether the stated raw materials were used [1]. These requirements place very great demands on the analysis of given samples. The analysis itself should be very fast, sufficiently sensitive and accurate, but also inexpensive. To meet these criteria, an application of electrochemical biosensors seems to be a good alternative.

Electrochemical biosensors combine two advantages: specificity of the enzyme to the given molecule and transfer of the biochemical signal to an electrochemical signal [2]. As a result, these biosensors are selective in establishing a specific substrate [3, 4]. By using these biosensors, it is possible to determine a large number of substances even in complex matrices.

Electrochemical biosensors often use redox enzymes during catalysis of substrate splitting reactions. Most used redox enzyme's are oxidases and dehydrogenases. There are several methods for establishing a substrates concentration. The most methods often used involve detecting hydrogen peroxide (a product of most oxidases) and nicotinamide adenine dinucleotide (NADH) (a product of dehydrogenases) resulting during the catalytic process. NADH oxidation on carbon electrodes requires high overvoltage (around 1.0 V). This is a highly unfavorable phenomenon, as the impact of interferents (e.g., uric acid, ascorbic acid, paracetamol) that are easily oxidized at a given overvoltage become most evident at such potentials. High overvoltage can be suppressed by using a so-called mediator [5–9] that enables the transfer of electrons between the enzymes active center, or the product of the enzyme reaction, and the electrodes surface. As the mechanism of NADH oxidation has not been fully explained, we have written it according to the generally recognized mechanism [10], as shown in the formula below (1):

(1) $$\begin{array}{c} \text{NADH}\overset{-{\text{e}}^{-}}{\to }{\text{NADH}}^{+∙}\overset{-{\text{H}}^{+}}{\to }{\text{NAD}}^{∙}\overset{-{\text{e}}^{-}}{⇌}{\text{NAD}}^{+}\\ \text{slow}\;\;\;\text{medium}\;\;\;\text{fast} \end{array}$$

The most important step in preparing a biosensor is that of enzyme immobilization. Should an inadequate procedure for enzyme entrapment be chosen, its denaturation, indirect inactivation, or washing from the electrode may occur. Many established immobilization techniques are currently used that include physical and chemical immobilization. Choice of enzyme immobilization method depends upon the properties of the enzyme, type of the mediator, conditions in which the biosensor is to work, and, last but not least, the physical properties of the analyte (or possibly the size of the molecules to be determined). Due to its simplicity, immobilization using electropolymerization [11–14] is one of the most commonly used techniques. Electropolymerization proceeds in a buffer solution that contains both a certain monomer and the enzyme itself which will be immobilized. A major advantage of this technique is the possibility to regulate the thickness of the membrane formed.

This article describes the preparation, optimization, and analytical properties of an enzyme biosensor prepared using the screen-printing technique, modified by rhodium dioxide, and containing glucose dehydrogenase immobilized in a layer of *m*-phenylenediamine (the main reason for this selection was price of the substance when compared with analogous *o-* or *p-*derivatives). The main advantage of this biosensor is that it works even at low input potentials, where contributions from other easily oxidizable or reducible molecules are negligible.

## 2. Experimental

### 2.1. Instrumentation

A modular electrochemical system, AUTOLAB, equipped with modules PGSTAT 30 and ECD (Ecochemie, Utrecht, Holand) was used in combination with corresponding software (GPES, Ecochemie).

The flow injection system consisted of a peristaltic pump (Minipuls 3, Gilson SA., France), a sample injection valve (ECOM, Ventil C, Czech Republic), and a self-constructed thin-layer electrochemical flow-through cell. The working electrode was fixed via rubber gaskets (thickness 0.6 mm) directly to the back plate of the thin layer cell. The reference electrode was Ag/AgCl/3M KCl (RE-6, BAS, USA), and the stainless steel back plate represented the counter electrode of the cell. The responses were evaluated using the peak heights (differences between background and response current of the analyte). Corresponding pH values were measured using a portable pH-meter (CPH 52 model, Elteca, Turnov, Czech Republic) equipped with a combined glass pH-sensor (OP-0808P, Radelkis, Budapest, Hungary). The measuring cell was calibrated using buffer solutions of the conventional activity scale.

### 2.2. Chemicals, Reagents, and Solutions

Glucose oxidase (EC 1.1.3.4. from *Aspergillus niger*, specific activity 198 U mg^−1^; GOx), glucose dehydrogenase (EC 1.1.1.47 from *Pseudomonas sp.*, specific activity 277  U mg^−1^; GDH), Nafion (5% m/m solution in lower aliphatic alcohols), nicotinamide adenine dinucleotide (NAD^+^) and its reduced form (NADH), rhodium dioxide, acetate cellulose (M *∼*37000 g moL^−1^), *m*-phenylenediamine, glutaraldehyde solution (GA, 50 wt. % in H_2_O), bovine serum albumin (BSA; 5% solution) and pyrrole (98% solution) were purchased from Aldrich. All other chemicals used for the preparation of buffer, stock, and standard solutions were of analytical reagent grade and purchased from Lachema (Brno, Czech Republic). Phosphate buffer was prepared by mixing aqueous solutions of sodium dihydrogenphosphate and disodium hydrogenphosphate (both 0.1 M) to achieve solutions of the required pH values. The glucose stock solution (2.5 g L^−1^) was prepared and diluted appropriately. Solutions of ascorbic acid and uric acid (both Aldrich, 50 mg L^−1^) were prepared immediately before use.

### 2.3. Electrode Preparation

Carbon ink (0.95 g, Gwent C50905D1, Pontypool, UK) and corresponding catalyst (0.05 g) were thoroughly mixed manually for 5 min and subsequently sonicated for 5 min. The resulting mixture was immediately used for the fabrication of electrodes. The working electrodes were prepared by screen-printing of modified ink onto an inert laser pre-etched ceramic support (113 × 166 × 0.635 mm, no. ADS96R, Coors Ceramics, Chattanooga, TN, USA). Thick layers of the modified carbon ink were formed by brushing the ink through an etched stencil (thickness 100 *μ*m, electrode printing area 105 mm^2^) with the aid of the spatula provided with the screen-printing device (SP-200, MPM, Franklin, MA, USA and/or UL 1505 A, Tesla, Czech Republic) onto the ceramic substrates. The resulting plates were dried at 60°C for 2 h.

### 2.4. Enzyme Immobilization

Several types of immobilization methods were tested with glucose oxidase, comprising entrapment in Nafion, cross-linking with glutaraldehyde, immobilization using cellulose acetate, and electropolymerization of pyrrole or *m*-phenylenediamine. Subsequently, a GDH enzyme together with cofactor NAD^+^ were immobilized using the best method, in terms of retaining enzyme activity, response time, sensitivity, and dynamic range of concentrations. 

#### 2.4.1. Entrapment in Nafion

An enzyme (GOx, 1 mg) was dissolved in 20 *μ*L of 0.1 M phosphate buffer (pH 7.5) and mixed with an equal amount of 0.05%, 0.5%, or 5% Nafion solution neutralized with ammonia to pH *∼*7. The resulting mixture (5 *μ*L) was applied directly onto the active area of the SPCE/RhO_2_ surface and air-dried for 30 min.

#### 2.4.2. Immobilization in Cellulose Acetate

An enzyme (GOx, 1 mg) was dissolved in 40 *μ*L of 0.1 M phosphate buffer (pH 7.5), and a volume of 3 *μ*L of this solution was applied onto the active area of the SPCE/RhO_2_ surface and air-dried. Subsequently, volumes of 3 *μ*L of cellulose acetate solution in acetone (0.05%, 0.5%, 1.5%, or 3.0%) were applied onto the aforementioned enzyme layer and dried for 5 min.

#### 2.4.3. Cross-Linking with Glutaraldehyde

Volumes of 5, 10 or 20 *μ*L of 5% glutaraldehyde (diluted with 0.1 M phosphate buffer, pH 7.5) were mixed with 1 *μ*L of 5% BSA and with 35 *μ*L, 30 *μ*L, or 20 *μ*L of the enzyme solution (1 mg of GOx in 0.1 M phosphate buffer, pH 7.5). After thorough mixing, a volume of 3 *μ*L was applied onto an SPCE/RhO_2_ and air-dried for 30 min. As a variant, cross-linking of the enzyme was also performed with GA vapor, whereby a volume of 3 *μ*L of the enzyme solution (1 mg of GOx in 40 *μ*L of 0.1 M phosphate buffer, pH 7.5) was applied onto the SPCE, air-dried (30 min), and then the SCPE/RhO_2_ so treated was enclosed overnight (17 hours) in a vial over 5% GA.

#### 2.4.4. Electropolymerization with Pyrrole

An enzyme solution (3 *μ*L, 1 mg of GOx in 40 *μ*L of 0.1 M phosphate buffer, pH 7.5) was applied onto an SPCE/RhO_2_. After drying for 30 min, the electrode was dipped into the 5 mM solution of pyrrole in 0.1 mM phosphate buffer, pH 6.0. Electropolymerization was performed at +0.75 V versus Ag/AgCl for 0.25, 0.5, 1.0, or 2.5 min, respectively. Finally, the electrode was washed with the phosphate buffer.

#### 2.4.5. Electropolymerization with m-Phenylenediamine—GOx

The procedure applied was similar to that described in the previous paragraph, but, concerning deposition time, the electrode was polarized in 5 mM *m*-phenylenediamine for 0.5, 1.0, 5, 10, or 20 min, respectively. Additionally, electropolymerization was performed at +0.75 V versus Ag/AgCl from 5 mM *m*-phenylenediamine GOx solution (10 mL containing 1 mg of GOx) for 5 min.

#### 2.4.6. Electropolymerization with m-Phenylenediamine—GDH

A volume of 3 *μ*L of NAD^+^ solution (3 mg in 40 *μ*L of 0.1 M pH 7.5 phosphate buffer) was applied onto the SPCE/RhO_2_ electrode surface. After drying, the surface was overlayered with GDH solution (3 *μ*L, 1 mg in 40 *μ*L of 0.1 M phosphate buffer) and dried for 45 min. An electrode was then dipped into the 5 mM solution (10 mL) of *m*-phenylenediamine in 0.1 mM phosphate buffer (pH 6.0) containing the remaining 37 *μ*L of NAD^+^ and 37 *μ*L of GDH solution, and it was left there for electropolymerization (5 min at +0.75 V versus Ag/AgCl). After washing with buffer, the electrode was prepared for measurements.

### 2.5. Procedure

Measurements were performed by DC amperometry using both flow injection and batch arrangements. All operational variables were optimized, that is, applied potential (from +0.6 to −0.3 V versus Ag/AgCl), pH of phosphate buffer (5–9), and flow rate (0.1–1.5 mL min^−1^). Responses were evaluated using the peak heights (differences between background and response current of the analyte). Injections of analyte were repeated at least three times.

### 2.6. Sample Processing

A sample of honey was prepared by dissolving the given amount of honey (3.4 g or 4.4 g of forest honey) in 50 mL of 0.1 M phosphate buffer of pH 7.5. Similarly, a sample of syrup was prepared, that is, 2.9 g of orange-flavored syrup was dissolved in 50 mL of 0.1 M phosphate buffer of pH 7.5. For analysis, 200 *μ*L of the samples thus prepared were always taken.

## 3. Results and Discussion

### 3.1. Effect of Enzyme Immobilization on Biosensor Response

Glucose oxidase was chosen as a test enzyme because of its stability and sensitivity to glucose [15]. For immobilizing glucose oxidase, the following methods and substances were used: immobilization in polymer—Nafion or cellulose acetate; immobilization using cross-linking—glutaraldehyde and BSA; electropolymerization—pyrrole or phenylenediamine. The entire study devoted to entrapment of the enzyme was performed in a batch arrangement in a cell with a volume of 10 mL. Selected key factors were monitored for each system: sensitivity, response time, and dynamic range.

#### 3.1.1. Entrapment in Nafion

**Figure 1:**
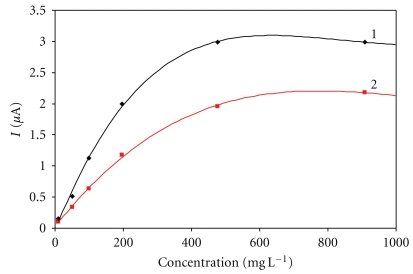
Immobilization using Nafion. Measurement condition: input potential −0.2 V (versus Ag/AgCl); 0.1 M phosphate buffer (pH 7.5); measured with SPCE/RhO_2_/GOx; analysis in a batch arrangement; concentration of Nafion: 1–0.5%, 2–5%.

Figure 1 shows calibration dependences obtained in immobilization of 0.5% GOx and 5% Nafion. The concentration of 0.05% was not sufficient to properly entrap the enzyme and the enzyme was shortly washed into the solution, which prevented further measurements. The dynamic ranges for Nafion concentrations of 0.5% and 5% were almost identical. The 0.5% Nafion, however, shows greater sensitivity to glucose and the response time here was the shortest, hovering around 28 s.

#### 3.1.2. Immobilization of GOx by Cross-Linking with Glutaraldehyde and BSA

**Figure 2:**
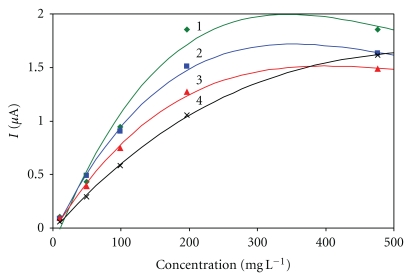
Immobilization using glutaraldehyde and BSA. Measurement condition: input potential −0.2 V (versus Ag/AgCl); 0.1 M phosphate buffer (pH 7.5); measured with SPCE/RhO_2_/GOx; analysis in a batch arrangement; concentration of glutaraldehyde: 1–0.625%, 2–1.25%, 3–2.5%, 4—immobilization with vapour of glutaraldehyde.

This immobilization procedure is very popular and well-proven for the design of enzyme electrochemical biosensors, and, therefore, it was included in this study. Concentrations of 0.625%, 1.25%, and 2.5% glutaraldehyde were compared here in a mixture with the enzyme. The possibility for enzyme immobilization using glutaraldehyde saturated vapors was examined as well (Figure 2). The response time was shortest in the case of enzyme immobilization using saturated vapors—30 s. While the response sensitivity to glucose decreased (poorer permeability of the analyte to the enzyme and poorer permeability of the metabolic product to the electrode's surface) with increasing thickness of the GA layer, the dynamic range of the setting increased at the same time.

#### 3.1.3. Immobilization Using Cellulose Acetate

For this study, solutions at concentration of 0.05%, 0.5%, 1.5%, and 3% of cellulose acetate in acetone were used. The first disadvantage of this method of biosensor preparation is the need to use the relatively volatile acetone, which vaporized very quickly while being pipetted and spread onto the electrodes surface. This resulted in an uneven distribution of the cellulose acetate layer. Acetone further dissolved the binder in carbon ink (of a resin type), which caused partial washing of the electrode.

**Figure 3:**
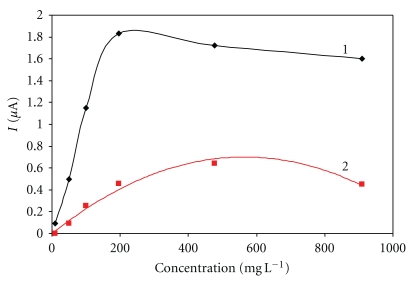
Immobilization using cellulose acetate. Measurement condition: input potential −0.2 V (versus Ag/AgCl); 0.1 M phosphate buffer (pH 7.5); measured with SPCE/RhO_2_/GOx; analysis in a batch arrangement; concentration of cellulose acetate in acetone: 1–1.5% and 2–0.5%.

Likewise in Nafion, the concentration of 0.05% was not sufficient to entrap properly the enzyme and no response to glucose was thus observed. By contrast, at the concentration of 3% the response to glucose was observed only for low concentrations of glucose up to 50 mg L^−1^; there was no increase in response above this concentration. The crucial disadvantage of this method, however, is its relatively long response time of 240 s. Another problem is the existence of a very narrow interval for usable concentrations of cellulose acetate for the enzyme immobilization within a range of 1% (Figure 3)—compared, for example, to Nafion with the choice of 0.5–5.0%.

#### 3.1.4. Immobilization by Pyrrole Electropolymerization

**Figure 4:**
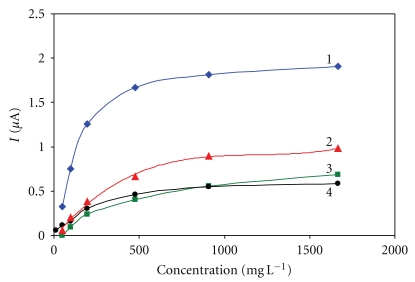
Immobilization using electropolymerization with pyrrole. Measurement condition: input potential −0.2 V (versus Ag/AgCl); 0.1 M phosphate buffer (pH 7.5); measured with SPCE/RhO_2_/GOx; analysis in a batch arrangement; time of electropolymerization 1–0.5 min, 2–1 min, 3–0.25 min, and 4–2.5 min.

Pyrrole was polymerized on the electrodes surface for periods of 0.25, 0.5, 1.0, and 2.5 min (Figure 4). The results show that for the period of 2.5 min, a very strong polypyrrole membrane is created which causes a slow transport of glucose molecules to the GOx enzyme and subsequently, the transport of H_2_O_2_ to the electrodes surface. This is evidenced by lower responses to glucose and longer response time. Another situation occurs for the period of 0.25 min. The enzyme is not sufficiently entrapped in this case, and, therefore, it is partially washed into the solution, which is again shown by very low responses. The best result was achieved using electropolymerization of pyrrole lasting 0.5 min. The responses are the highest here and the response time of 35 s is also acceptable.

#### 3.1.5. Immobilization Using Electropolymerization with *m*-Phenylenediamine

**Figure 5:**
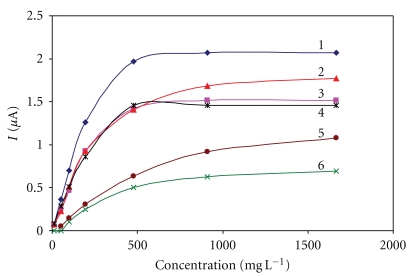
Immobilization using electropolymerization with *m*-phenylenediamine. Measurement condition: input potential −0.2 V (versus Ag/AgCl); 0.1 M phosphate buffer (pH 7.5); measured with SPCE/RhO_2_/GOx; analysis in a batch arrangement; time of electropolymerization 1–1 min, 2–10 min, 3–5 min, 4–0.5 min, 5–5 min with addition of 1 mg GOx to electropolymerization mixture, and 6–20 min.

The *m*-phenylenediamine was polymerized onto the electrodes surface for periods of 0.5, 1, 5, 10, and 20 min. Furthermore, GOx was incorporated directly into the phosphate buffer solution with *m*-phenylenediamine and the electropolymerization was performed for 5 min.  Figure 5 shows that the best response to glucose was achieved when the *m*-phenylenediamine was electropolymerized for 1 min. Shorter times were insufficient to entrap the enzyme into the polymeric membrane. Longer times, however, created a thicker membrane which slowed the processes, transporting the analyte to the enzyme and the metabolite to the electrodes surface, which was similar to the situation for pyrrole.

In the case of electropolymerization with *m*-phenylenediamine together with the enzyme directly in a phosphate buffer solution, the sensitivity was almost the lowest and the response time was relatively long (100 s). However the dynamic range was greatest in this case

#### 3.1.6. Comparison of the Immobilization Techniques

Table 1 compares the various methods of immobilization. Ideally, a biosensor should have the shortest-possible response time, the largest dynamic range of concentrations, and highly sensitive responses to the given analyte. In practice, however, it is necessary to compromise and to favour one parameter over another according to the determination requirements. Since all the immobilizations listed show rather sensitive responses to glucose, the decisive criteria are response time and dynamic range. The most appropriate method can therefore, be considered the electropolymerization with *m*-phenylenediamine, which was used for immobilization of the glucose dehydrogenase enzyme.

### 3.2. Determination of Glucose by Glucose Dehydrogenase

From the methods of immobilization examined, that one using electropolymerization with *m*-phenylenediamine was selected for preparation of the given biosensor. When working with dehydrogenases, great emphasis must be given to correctly executing the immobilization, because not only the enzymes but also their cofactors (NAD^+^ or NADP^+^) are immobilized. These cofactors are soluble in aqueous solutions and thus they wash rapidly into the solution, and especially when using flow analysis. The entire procedure for electrode preparation is described in Section2.4.6 (while Section 3.1.5 stated that the best response to glucose was reached where *m*-phenylenediamine was electropolymerized for 1 min, an electropolymerization time of 5 min was chosen here due to better entrapment of the NAD^+^ cofactor.)

#### 3.2.1. Effect of the Potential on the Biosensor Response

**Figure 6:**
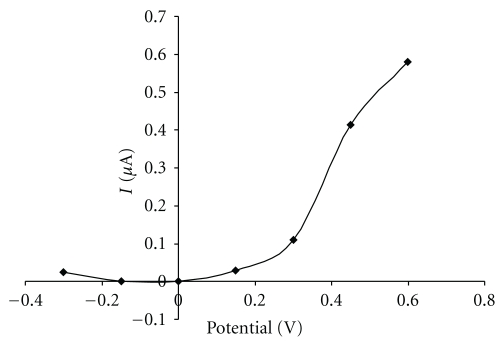
Effect of potential on the biosensor response. Measurement condition: glucose concentration 1000 mg L^−1^; batch volume 200 *μ*L; pH of the supporting electrolyte 7.5; flow rate 0.2 mL min^−1^; measured on SPCE/RhO_2_/GDH; analysis in a flow arrangement.

Input potential is one of the most important parameters in the amperometric determination of analytes since its choice affects the selectivity of the given biosensor.  Figure 6 shows the dependence of response on the operating potential (dependence of the peak size on the potential was observed in the range of −0.3 to +0.6 V versus Ag/AgCl in 0.15 V intervals). As is visible there, oxidation starts at around +0.15 V and the response increases with the increasing potential. Oxidation is also observed in the vicinity of −0.3 V, but this response is very low and, therefore, unsuitable for determination of glucose. In the range of −0.2 V to +0.1 V, the biosensor records no catalytic activity. As this shows, the most appropriate area for determination of glucose is in the range of +0.15 to +0.6 V (taking into consideration the effect of interferents).

#### 3.2.2. Effect of Interferents on the Biosensor Response

**Figure 7:**
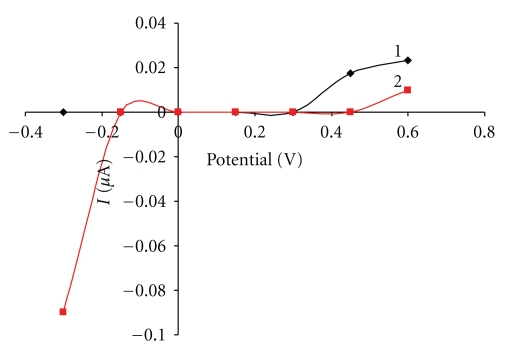
Effect of interferents on the biosensor response. Measurement condition: ascorbic acid, and uric acid concentrations 10 mg L^−1^; batch volume 200 *μ*L; pH of the supporting electrolyte 7.5; flow rate 0.2 mL min^−1^; measured on SPCE/RhO_2_/GDH; analysis in a flow arrangement; 1—ascorbic acid, 2—uric acid.

There can be many interfering substances in the samples (such as blood and food). The most important interferents include ascorbic acid, uric acid and paracetamol. It has been observed that all of these are electroactive at the applied potential of +0.5 V, but, in the potential window of −0.2 to +0.45 V, their responses are negligible (Figure 7). For this reason, potentials in the given range were chosen for further work.

#### 3.2.3. Effect of Flow Rate and pH on the Biosensor Response

**Figure 8:**
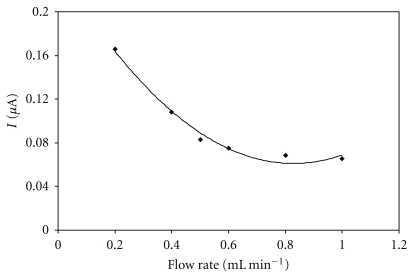
Effect of flow rate on the biosensor response. Measurement condition: glucose concentration 1000 mg L^−1^; batch volume 200 *μ*L; input potential 0.45 V (versus Ag/AgCl); pH of the supporting electrolyte 7.5; measured on SPCE/RhO_2_/GDH; analysis in a flow arrangement.

Flow rate also belongs among the very important parameters that must be optimized. It was done in the range of 0.1 mL min^−1^ to 1 mL min^−1^.  Figure 8 shows that the size of the response decreases with an increasing flow rate. This is due to the fact that if the flow rate is too high, the NADH+ on the electrode is not fast enough to react. On the other hand, at low flow rates, the biosensors response is unstable (decrease of the response by 20% over three determinations). This response instability was probably caused by passivation of the electrode's surface. For this reason, a flow rate ranging from 0.4 to 0.6 mL min^−1^ seemed ideal. For other measurements, the flow rate of 0.5 mL min^−1^ was chosen. That seems to be a good compromise between buffer consumption, response stability, and speed of the experiment.

Optimization of pH was carried out in the range of 5 to 9. Stable responses were observed at all measured pH values and the highest was achieved at pH 8, where at the same time the maximum enzyme activity is seen. For further work, the pH of 7.5 was chosen because the given pH is close to the physiological pH and that is optimal for the determination of biological substances in food and especially in clinical samples.

#### 3.2.4. Biosensor Response to Glucose

Calibration dependences were measured at two different potentials (+0.35 V and +0.45 V). At the potential of +0.35 V, the biosensor showed lower responses, but the dynamic range was greater than at the potential of +0.45 V. A big advantage is that at the input potential of +0.35 V, the effects of interferents are much more suppressed. The proposed biosensor retained its activity after more than 50 injections. No loss of the original signal was achieved after 1 month, when stored at 6°C in the refrigerator.

### 3.3. Real Samples

Honey and syrup samples were used as real analytes. Measurement was performed under these optimized conditions: input potential +0.35 V; batch volume 200 *μ*L; 0.1 M phosphate buffer pH 7.5; flow rate 0.5 mL min^−1^in a three-electrode arrangement in the presence of SPCE/RhO_2_/GDH, where the enzyme was entrapped by *m*-phenylenediamine. The determined concentrations are shown in Table 2.

The amperometric determination with SPCE/RhO_2_/GOx was used as a reference method (carbon printed electrode modified by glucose oxidase and rhodium oxide—the enzyme immobilized by Nafion). Measurement conditions: −0.2 V (versus Ag/AgCl); phosphate buffer pH 7.5; flow rate 0.2 mL min^−1^; batch volume 50 *μ*L.

## 4. Conclusion

A biosensor containing rhodium dioxide and glucose dehydrogenase enzyme was prepared using the screen-printing technique. Various methods of enzyme immobilization were tested, among which *m*-phenylenediamine electropolymerization proved the best. It excelled with its response time, sensitivity, and signal stability. The enzyme biosensor was optimized and tested in model glucose samples and also applied to analyze real samples (honey, syrup).

Good results in the determination of glucose in real samples indicate, among other things, that the biosensor was not affected by any complicated sample matrix (ascorbic acid and other oxidizable substances) and has prospects for use also for similar applications in the food industry and clinical practice.

## Figures and Tables

**Figure 9:**
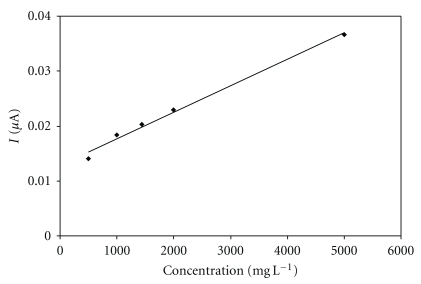
Biosensor response to glucose at input potential +0.35 V. Measurement condition: batch volume 200 *μ*L; input potential 0.35 V (versus Ag/AgCl); pH of the supporting electrolyte 7.5; flow rate 0.5 mL min^−1^; measured on SPCE/RhO_2_/GDH; analysis in a flow arrangement; regression equation: *y* = 5.00 × 10^−6^ *x* + 0.0129, *R* ^2^ = 0.991.

**Table 1:** Comparison of individual immobilization methods and their parameters.

Type of immobilization	Response* (*μ*A)	Linearity (mg L^−1^)	Response time (s)
Nafion (0.5%)	1.991	10–200	120
Glutaraldehyde vapors	1.054	10–200	30
Cellulose acetate (1.5%)	1.831	10–200	240
Pyrrole (0.5 min)	1.255	50–250	35
*m*-phenylenediamine (1 min)	1.260	10–500	25

* Measured at glucose concentration of 200 mg L^−1^.

**Table 2:** Determination of glucose in real sample using SPCE/RhO_2_/GDH.

		Proposed method		Reference method		
Sample	*n*	$\bar{x}\pm R\;\left[\%\right]$	*n*	$\bar{x}\pm R\;\left[\%\right]$	*u*	*u*_crit_
Honey	4	33.84 ± 5.63	4	33.97 ± 2.16	0.017	0.406
Syrup	4	26.06 ± 4.70	4	24.31 ± 4.74	0.185	0.406

*n*: number of measurements; *x*: arithmetic mean; *R*: range; *u*_crit_ and *u*: critical and calculated values of Lord's test (selected probability—95%).
